# Spatial and temporal characteristics analysis and prediction model of PM_2.5_ concentration based on SpatioTemporal-Informer model

**DOI:** 10.1371/journal.pone.0287423

**Published:** 2023-06-23

**Authors:** Zhanfei Ma, Wenli Luo, Jing Jiang, Bisheng Wang, Ziyuan Ma, Jixiang Lin, Dongxiang Liu

**Affiliations:** 1 School of Information Science and Technology, Baotou Teachers’ College, Baotou, Inner Mongolia, China; 2 School of Information Engineering, Inner Mongolia University of Science and Technology, Baotou, Inner Mongolia, China; 3 College of Mechanical and Electrical Engineering, Sichuan Agricultural University, Ya ’an, Sichuan, China; UCSI University Kuala Lumpur Campus: UCSI University, MALAYSIA

## Abstract

The primary cause of hazy weather is PM_2.5_, and forecasting PM_2.5_ concentrations can aid in managing and preventing hazy weather. This paper proposes a novel spatiotemporal prediction model called SpatioTemporal-Informer (ST-Informer) in response to the shortcomings of spatiotemporal prediction models commonly used in studies for long-input series prediction. The ST-Informer model implements parallel computation of long correlations and adds an independent spatiotemporal embedding layer to the original Informer model. The spatiotemporal embedding layer captures the complex dynamic spatiotemporal correlations among the input information. In addition, the ProbSpare Self-Attention mechanism in this model can focus on extracting important contextual information of spatiotemporal data. The ST-Informer model uses weather and air pollutant concentration data from numerous stations as its input data. The outcomes of the trials indicate that (1) The ST-Informer model can sharply capture the peaks and sudden changes in PM_2.5_ concentrations. (2) Compared to the current models, the ST-Informer model shows better prediction performance while maintaining high-efficiency prediction $\left(MAE\approx 7.50\mu g/m^{3},RMSE\approx 4.31\mu g/m^{3},R^{2}\approx 0.88\right)$. (3) The ST-Informer model has universal applicability, and the model was applied to the concentration of other pollutants prediction with good results.

## Introduction

China’s rapid industrialization and urbanization are worsening urban air pollution, which has detrimental effects on individuals’ lives, health, and economic activity [1–3]. To reduce air pollution, relevant departments have proposed important initiatives such as haze control and improvement of environmental quality [4]. As a significant component of atmospheric pollutants, PM_2.5_ is a major factor in causing hazy weather, reducing visibility, and affecting traffic safety. Long-term exposure to high PM_2.5_ concentrations can result in cancer of the lungs, cardiovascular disease, respiratory disorders, and other health significantly impacts youngsters, the elderly, and those with heart or lung disease. In addition, The development of aquatic organisms and plants can also be impacted by high PM_2.5_ concentrations that land in soil and water bodies. Therefore, accurate prediction of PM_2.5_ concentration is of great practical importance for national air pollution control and risk avoidance. However, PM_2.5_ concentrations are often influenced by various external factors and have complex time and space dependence [5]. Therefore, the development of highly precise and effective PM_2.5_ prediction systems is still an urgent scientific issue.

With the advancement of science and technology, monitoring tools, including weather observation stations, air quality monitoring stations, and meteorological satellites, have started to be extensively dispersed in diverse locations. Because the massive amount of air pollutant concentration data and meteorological data collected by this equipment provides a large amount of data support for air quality prediction and meteorological research, PM_2.5_ concentration prediction models built on data-driven models are receiving increasing attention from research scholars. Previous studies have used statistical models to predict PM_2.5_ concentrations, such as Auto-regressive Moving Average (ARMA) model [6, 7] and Auto-regressive Integrated Moving Average (ARIMA) model [8, 9], which are traditionally spartan in structure, less computationally intensive, and can effectively describe the variability of time series; however, these models tend to oversimplify the complexities between PM_2.5_ and other air pollutant concentrations non-linear relationships, which are prone to bias in the face of non-linear relationships. The development of pollutant concentration prediction models based on machine learning algorithms has been rapid in recent years [10], and the nonlinear regression prediction performance of these models is better than that of traditional statistical methods, and commonly used models include Support Vector Regression (SVR) [11], random forest models [12], and Artificial Neural Networks (ANN) [13–15]. In addition, artificial neural networks have strong adaptive and robust properties and show excellent performance in solving prediction problems, especially Recurrent Neural Network (RNN); RNN and their variants Long Short Term Memory Network (LSTM) in processing and are commonly used for pollutant concentration prediction in different time spans [16, 17]. Since various factors influence the concentration of pollutants, the data’s long-term time dependence and dynamic spatial correlation need to be considered when constructing prediction models. There are studies to construct spatiotemporal prediction models by fusing different neural network structures, such as Convolutional Neural Network and Long Short Term Memory Network (CNN-LSTM) model; CNN-LSTM model by CNN layer Convolutional operations are performed on the input data, and the spatial feature information extracted from it is passed to the LSTM layer to capture the dynamic changes and long-term dependencies in the spatiotemporal sequence data [18, 19]. In addition, there are Graph Convolutional networks and Long Short Term Memory Networks (GC-LSTM) model [20], Long Short Term Memory-Fully Connected Neural Network (LSTM-FC) model [21], and other combined spatiotemporal prediction models that are more comprehensive in dealing with spatiotemporal data with multidimensional features. However, due to the limitations of convolutional filters and the shortcomings of LSTM, which cannot be computed in parallel, these models cannot adequately extract the long-term and complex correlations of spatiotemporal data when the input data is extended. Some studies have compensated for these shortcomings in the above models by introducing the Attention mechanism [22, 23], the addition of the Attention mechanism can be used to focus on extracting important information in the context, but their inherent characteristics limit their predictive power for long spatiotemporal sequences.

Vaswani et al. [24] proposed the Transformer model, which is a model with a new architecture built entirely based on the Attention mechanism and achieves higher parallelism, which has achieved excellent results in Natural Language Processing (NLP) [25], Computer Vision (CV), and other fields. Considering that the model allows parallel processing and temporal feature extraction for time-series data, many studies have applied the Transformer model to time-series data prediction tasks [26]. For example, Li S et al. [27] proposed an Enhanced Local Transformer (ELM) model that enhances the Transformer’s local and context-aware capabilities in processing time series data by introducing adaptive location coding and spatially aware attention mechanisms, and the ELM model achieves better performance on various time series prediction tasks. In addition, Zhou et al. [28] developed an Informer model, which improves the traditional Transformer model by introducing new structures and mechanisms to learn nonsmooth and long-term time dependence and has higher efficiency and accuracy when dealing with long series time series data [29]. However, the Informer model focuses only on the "temporal attention" between learning time steps and ignores the complex spatial relationships between variables. Therefore, to predict PM_2.5_ concentrations with spatiotemporal characteristics more accurately and effectively, this paper proposes a spatiotemporal prediction model: the SpatioTemporal-Informer (ST-Informer) model. The ST-Informer model has an independent spatiotemporal embedding layer, which enables a comprehensive analysis of the spatiotemporal correlation of the input data. Secondly, considering that meteorological characteristics and spatial characteristics of data can affect the prediction of pollutant concentrations [30], auxiliary data such as meteorological data and atmospheric pollutant concentration data from multiple stations were integrated into predicting PM_2.5_ concentrations to improve the performance of the model. The results show that the ST-Informer model has spatiotemporal prediction capability and high prediction accuracy. The main contributions of this paper are as follows.

1. In this paper, the influence of multi-source data is considered comprehensively, and adjacent stations’ meteorological factors and pollutant concentration factors affect the PM_2.5_ concentration prediction at the central station. Therefore, the study selects meteorological data and pollutant concentration data from multiple stations as input data.
2. Due to anomalies and missing data collected, the ST-Informer model is relatively sensitive to outliers and noise in the input sequence, and the presence of many outliers or noise will affect the prediction results of this model. Therefore, this paper performs complete pre-processing work on the data. Then, Pearson correlation coefficients were used to analyze the strength of the data’s temporal and spatial correlations to ensure the input data’s strong correlation as the ST-Informer prediction model in PM_2.5_ prediction and enhance the model’s prediction performance.
3. The independent temporal embedding layer and spatial embedding layer in the ST-Informer model proposed in this paper are used to process the input data and capture the complex spatiotemporal characteristics of the data. As well as the ProbSpare Self-Attention mechanism, which is unique to this model, concentrates on extracting information of data across time, space, and multiple dimensions. The model can analyze the spatiotemporal correlation of data more comprehensively. Its prediction effect is better than LSTM, Transformer, and Informer models through the comparative analysis of the results of three evaluation indexes. And the ST-Informer model is applied to the prediction of other pollutant concentrations, which proves its universality.

## Materials

### Description of the data

**Fig 1:**
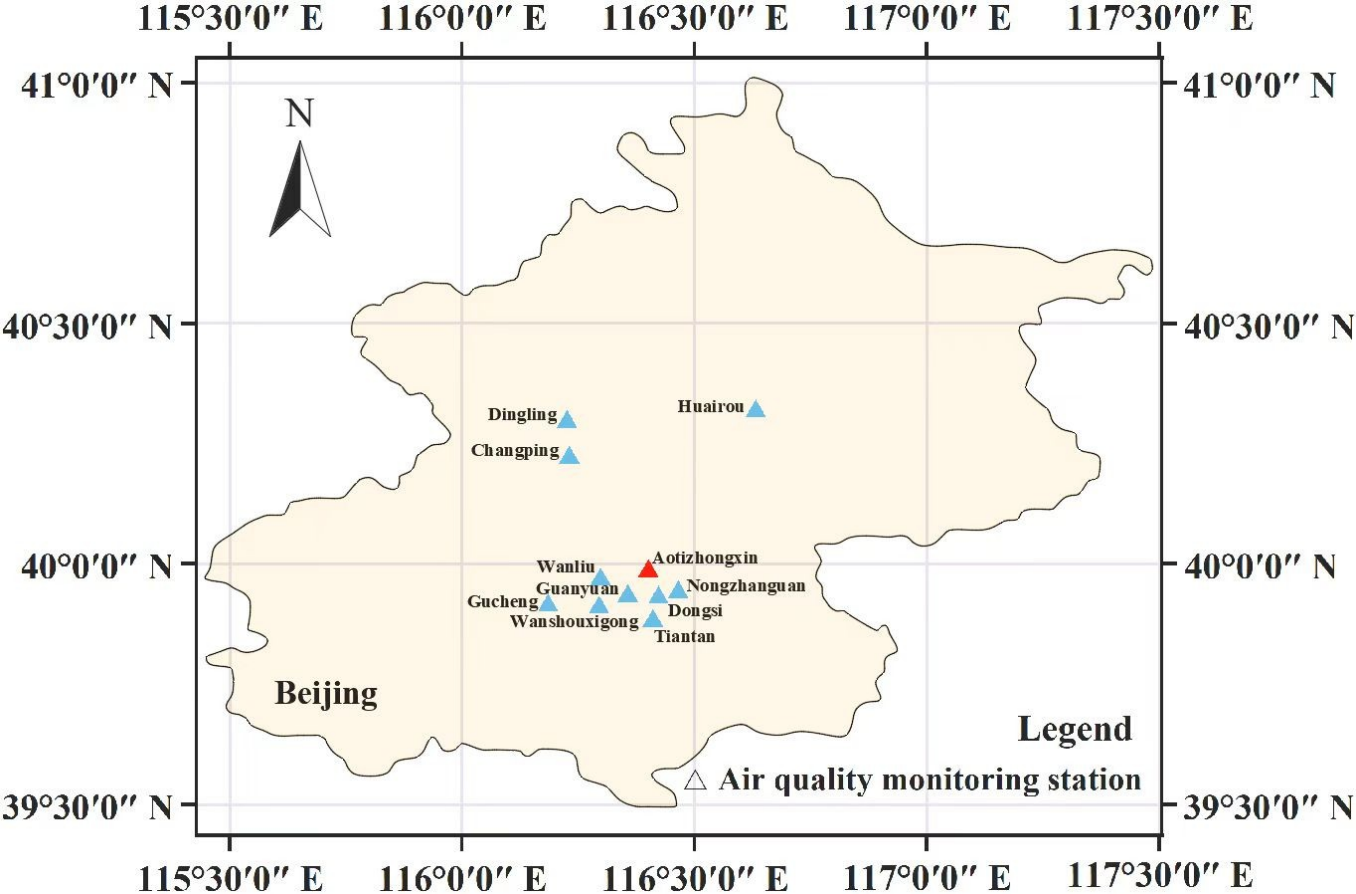
Coordinates of air quality monitoring stations. Location map of each air quality monitoring station in Beijing.

Because of the rapid economic growth, dense population, and industrialization of the Beijing-Tianjin-Hebei area, in this paper, we take the forecast of PM_2.5_ concentration in Beijing as an example. The U.S. Embassy in Beijing, China provided the hourly meteorological and pollutant concentration data from 11 air quality monitoring stations in Beijing that were collected between March 1, 2013, at 00:00 and February 28, 2017, at 23:00 (http://archive.ics.uci.edu/ml/datasets/Beijing+Multi-Site+Air-Quality+Data). Fig 1 depicts the locations of each station, and Table 1 lists the latitude and longitude of each station. The Aotizhongxin Center’s air quality monitoring station, which is close to all the other sites, was chosen as the central site. The 10 other sites, including Changping, that had strong correlations with the central site were then identified by looking at the spatial and temporal correlation of the data. As shown in Table 2, the pollutant concentration factors include the following 6 variables: PM_2.5_, PM_10_, SO_2_, NO_2_, CO, and O_3_. The weather variables primarily consist of 6 variables: temperature, atmospheric pressure, dew-point temperature, rainfall capacity, wind direction, and wind speed.

**Table 1:** Name of air quality monitoring station.

Station Name	longitude	latitude
Aotizhongxin	116°24’3.028"	39°59’6.562"
Changping	116°13’51.722"	40°13’15.427"
Dingling	116°13’32.732"	40°17’41.435"
Dongsi	116°24’55.862"	39°55’46.423"
Guanyuan	116°21’24.548"	39°55’57.986"
Gucheng	116°11’6.130"	39°54’47.041"
Huairou	116°37’55.106"	40°19’1.211"
Nongzhanguan	116°27’54.468"	39°56’25.872"
Tiantan	116°24’38.984"	39°52’54.887"
Wanliu	116°17’53.063"	39°58’1.758"
Wanshouxigong	116°17’40.420"	39°54’34.171"
Aotizhongxin	116°24’3.028"	39°59’6.562"
Changping	116°13’51.722"	40°13’15.427"
Dingling	116°13’32.732"	40°17’41.435"
Dongsi	116°24’55.862"	39°55’46.423"
Guanyuan	116°21’24.548"	39°55’57.986"
Gucheng	116°11’6.130"	39°54’47.041"
Huairou	116°37’55.106"	40°19’1.211"
Nongzhanguan	116°27’54.468"	39°56’25.872"
Tiantan	116°24’38.984"	39°52’54.887"
Wanliu	116°17’53.063"	39°58’1.758"
Wanshouxigong	116°17’40.420"	39°54’34.171"

**Table 2:** Attributes of the correlation factors input when the model is making predictions.

data name	data type	unit
PM_2.5_	PM_2.5_ Treatment serum concentration	*μm*/*m*^3^
PM_10_	PM_10_ Treatment serum concentration	*μm*/*m*^3^
SO_2_	SO_2_ Treatment serum concentration	*μm*/*m*^3^
NO_2_	NO_2_ Treatment serum concentration	*μm*/*m*^3^
CO	CO Treatment serum concentration	*μm*/*m*^3^
O_3_	O_3_ Treatment serum concentration	*μm*/*m*^3^
TEMP	temperature	°C
PRES	atmospheric pressure	*pa*
DEWP	dew-point temperature	°C
RAIN	rainfall capacity	*mm*
WD	wind direction	orientation
WSPM	wind speed	*m*/*s*

### Data preprocessing

The factors predicted in this study include pollutant concentrations (PM_2.5_, PM_10_, SO_2_, NO_2_, CO, O_3_) and meteorological factors (temperature, atmospheric pressure, dew-point temperature, rainfall capacity, wind direction, wind speed). Before using this dataset as input data for PM_2.5_ concentration prediction, data pre-processing is performed. The data preprocessing mainly includes outlier testing, missing value filling, and data normalization.

1. Test for outliers

**Fig 2:**
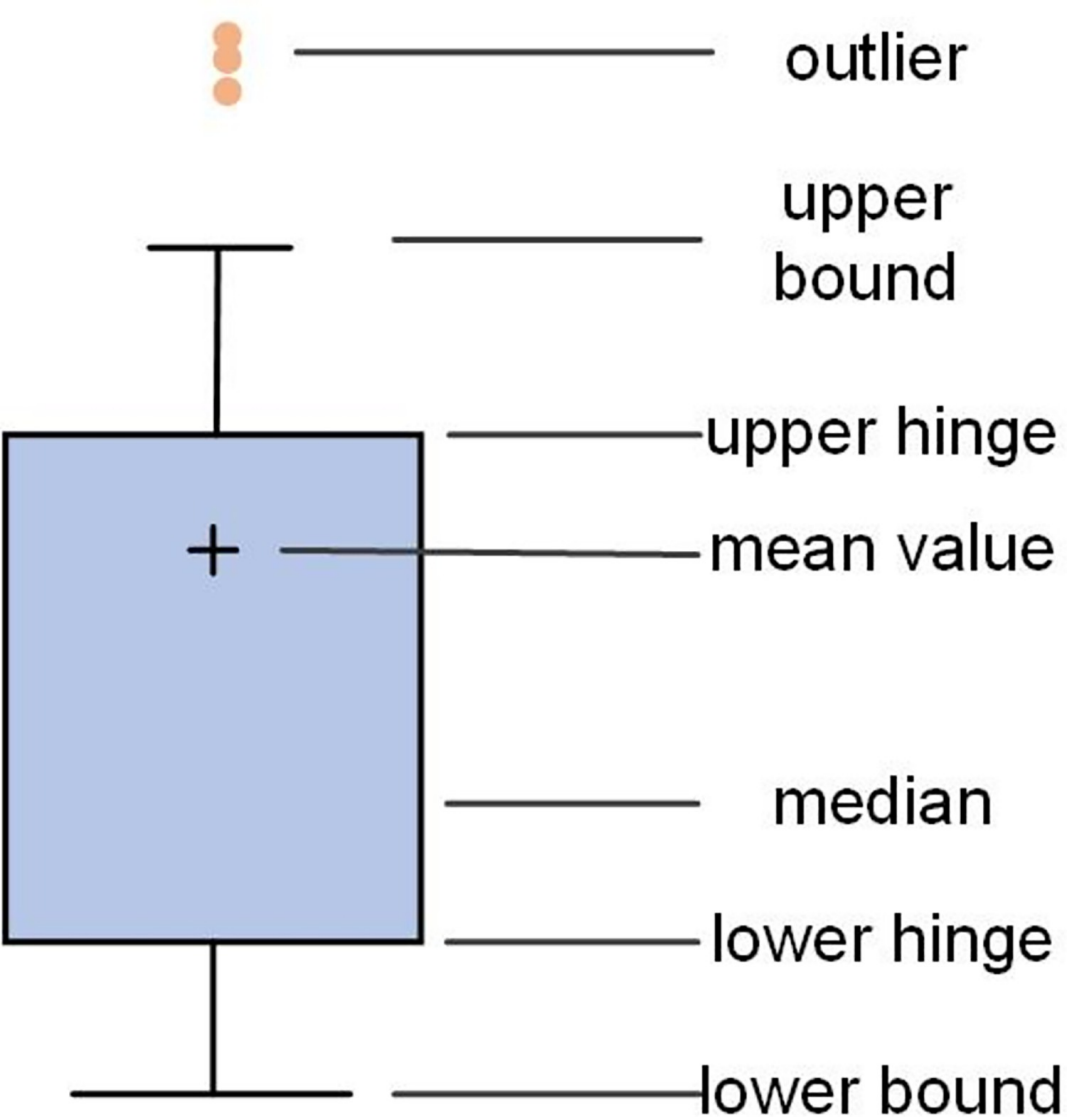
Structure of the box line diagram. A boxplot is a statistical graph showing the distribution of one or more sets of continuous quantitative data, the first quartile, the median, and the third quartile.

In this paper, outliers are examined using the principle of box plot, which is a statistical graph used to display the dispersion of a set of data [31], and its structure is shown in Fig 2. The box plot provides a criterion for identifying outliers, i.e., a value greater than or less than the upper and lower bounds set by the box plot is considered an outlier.

**Fig 3:**
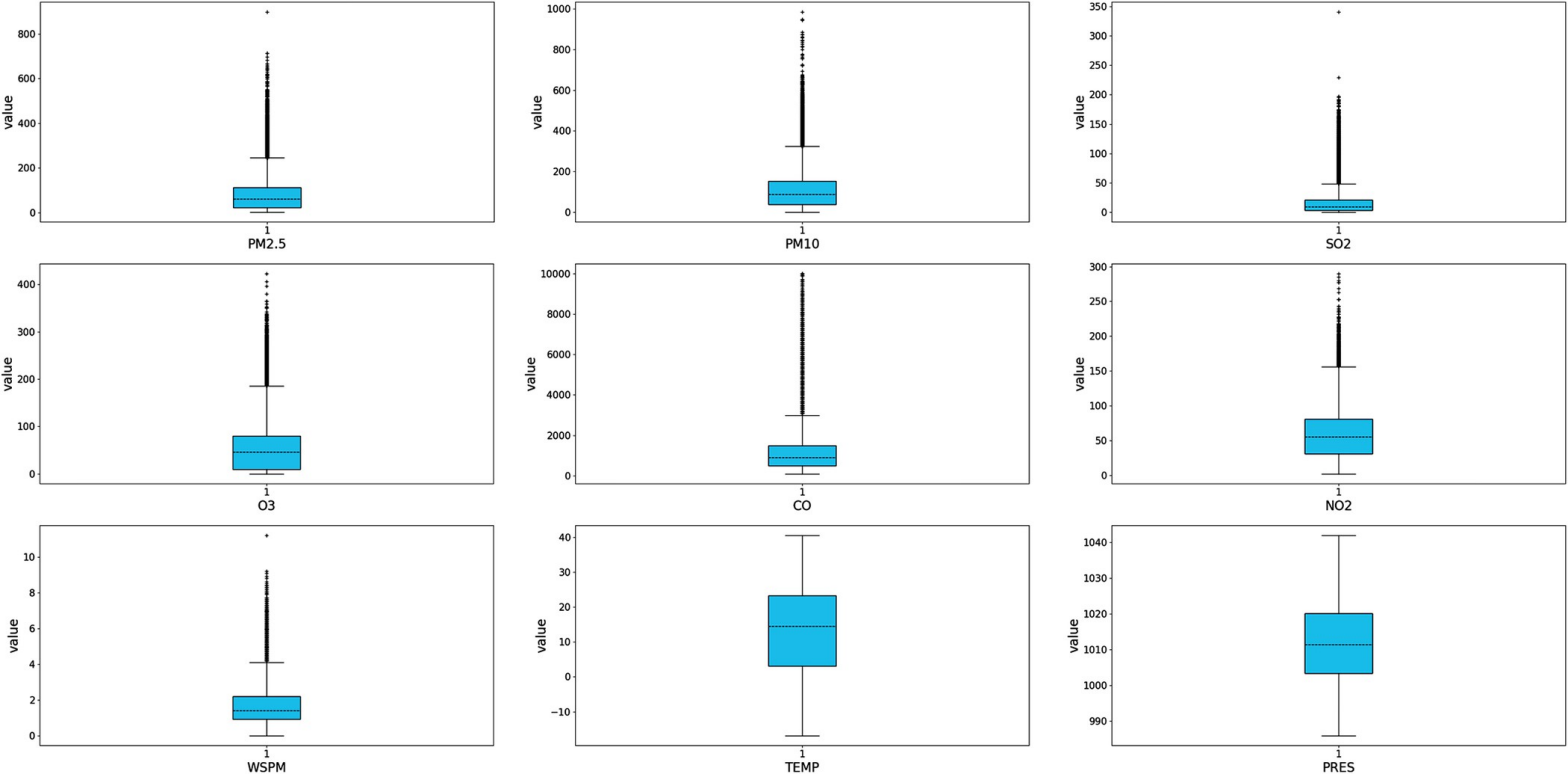
The resulting graph of outliers is tested using a boxplot. Taking the air quality monitoring station of the Aotizhongxin as an example, this research used the boxplot principle to test the outliers of the collected historical data.

The historical data collected from each air quality monitoring station were first examined for outliers. Taking the Aotizhongxin station as an example, the image of the meteorological data and air pollutant concentration data collected at this station after processing by the box-line diagram principle is shown in Fig 3. The examined outliers are then marked as missing values.

2. Missing value padding

**Fig 4:**
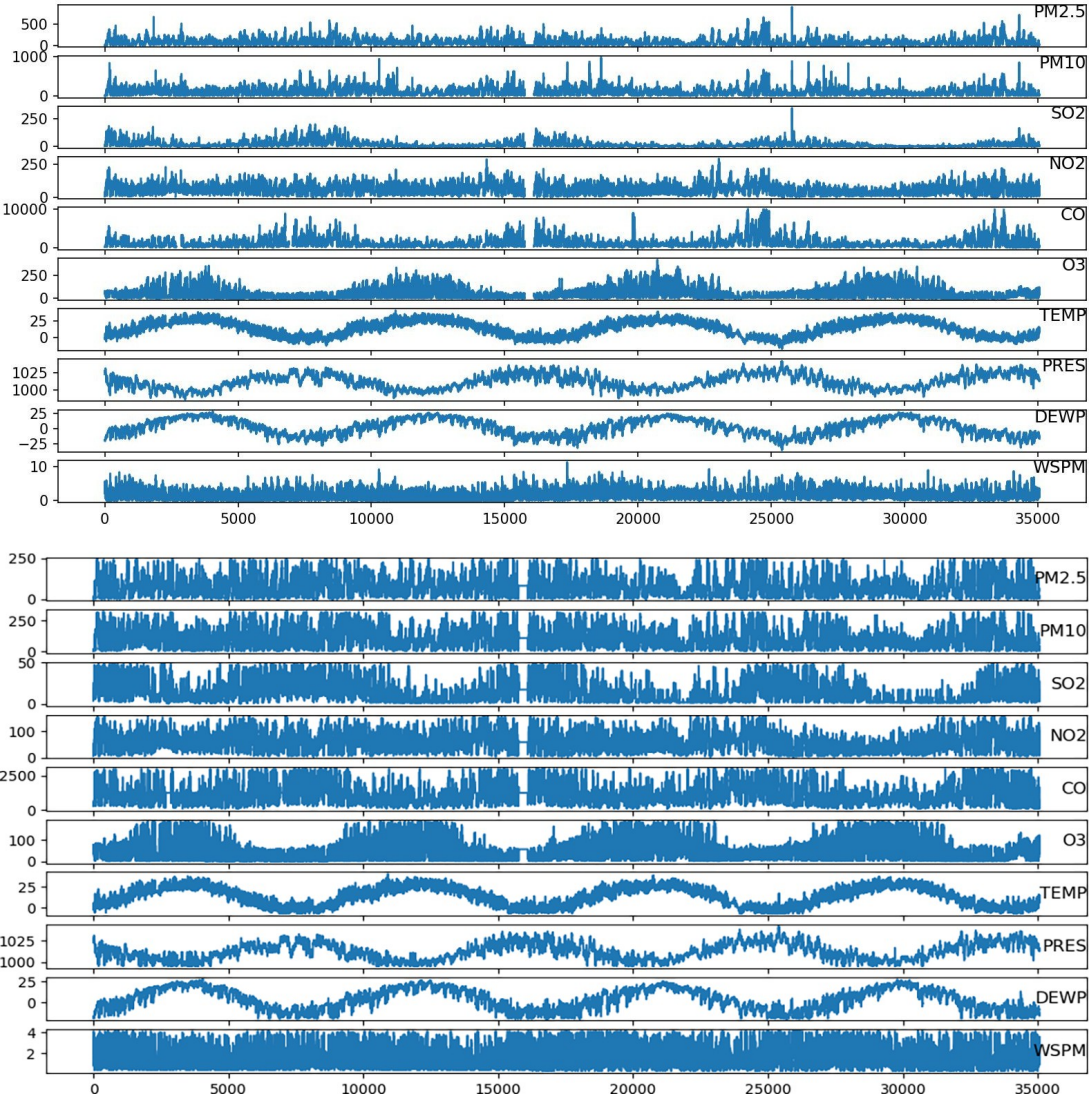
Pre-processed data visualization. Comparison of meteorological data and air pollutant concentration data before and after data preprocessing at the central station. (a) Visualization of unprocessed data. (b) Visualization of pre-processed data.

The method for filling in the missing values in this paper is the K Nearest Neighbor Classification (KNN) algorithm [32], which estimates the missing data by identifying K sample points adjacent to the missing values and using the average of these K sample points. As shown in Fig 4, a comparison graph of unprocessed and processed historical data from the air quality monitoring sites in the Aotizhongxin clearly shows that the outliers are significantly reduced and the data are more stable after being processed using the KNN algorithm.

3. Normalization of data

The data are then normalized [33], which is treating each feature equally by removing the variability of values and units between feature parameters and normalizing the initial feature data to increase prediction accuracy. The normalization expression is as follows:

$$x_{i}=\frac{x_{i}-min}{max-min}$$ (1)

## Methods

### Spatiotemporal correlation

Because meteorological elements like the wind impact air pollutants, the concentration of pollutants at one site can impact the concentration of pollutants at nearby locations. Because of this, spatial and temporal correlations between the data are extracted when making predictions of air pollutant concentrations based on data on pollutant concentrations and meteorological data from the central and nearby locations.

To derive elements of spatiotemporal correlation, use the Pearson correlation coefficient. The Pearson correlation coefficient is a statistic used to determine how closely two factors are correlated linearly and to compare traits and categories. A higher absolute number denotes a stronger correlation between the two variables, and the Pearson correlation coefficient ranges from -1 to 1. The formula for calculating the Pearson correlation value is:

$$\begin{array}{l} \rho_{X,Y}=\frac{cov(X,Y)}{\sigma_{X}\sigma_{Y}}=\frac{E(\left(X-\mu_{X})(Y-\mu_{Y}\right))}{\sigma_{X}\sigma_{Y}}\\ =\frac{E(XY)-E(X)E(Y)}{\sqrt{E(X^{2})-E^{2}(X)}\sqrt{E(Y^{2})-E^{2}(Y)}} \end{array}$$ (2)

Where *X* and *Y* represent the two variables, *cov*(*X*,*Y*) represents the covariance between the two variables, and σ_*x*_σ_*y*_ represents the standard deviation of the two variables.

**Fig 5:**
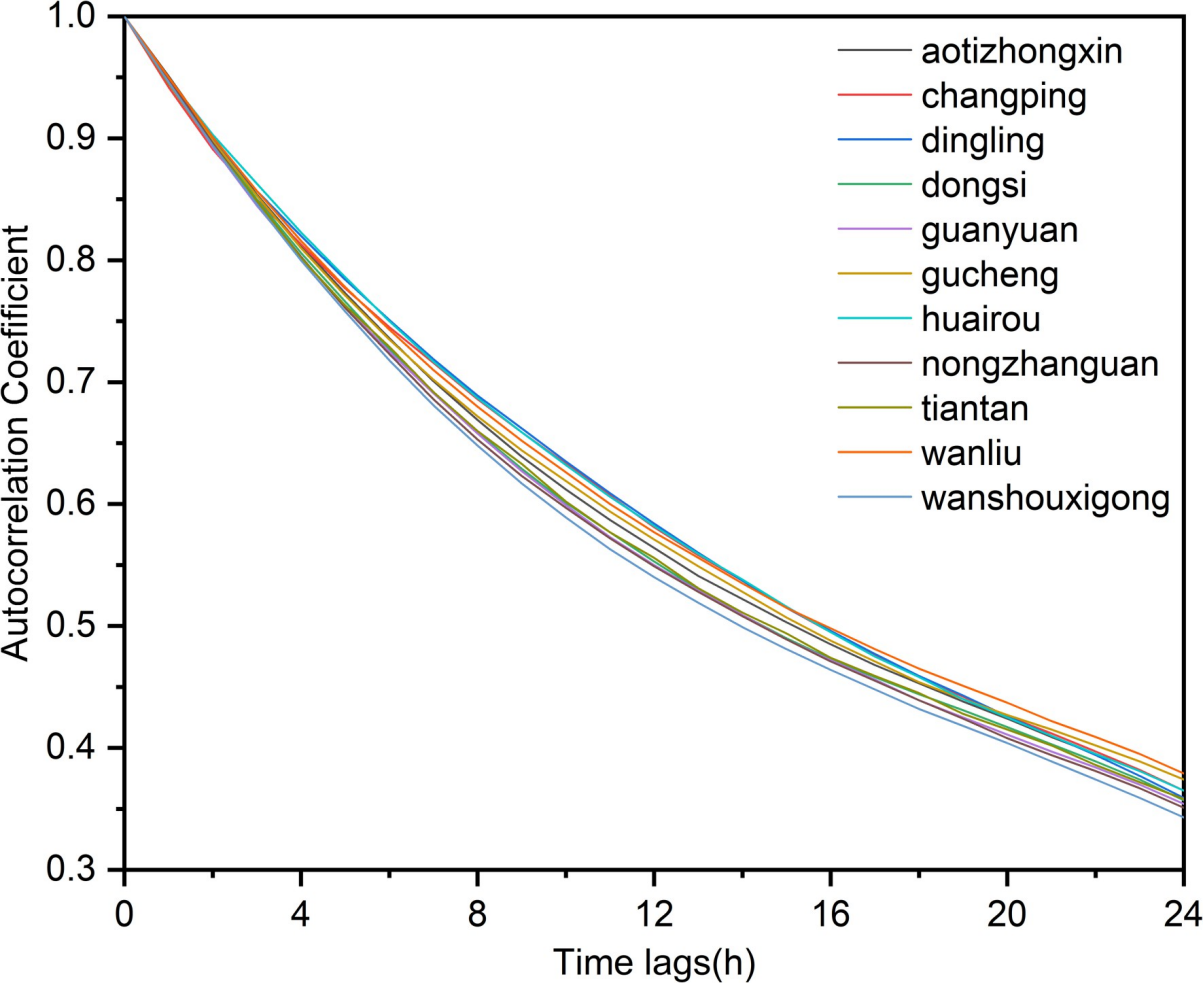
Time autocorrelation analysis. The autocorrelation coefficient of each station changes with time delay of 0–24 hours.

In terms of extracting temporal correlation analysis, the Pearson correlation coefficient was used to assess the degree of temporal correlation, with *X* and *Y* in the Pearson correlation coefficient formula representing the values of historical data before and after a specific time interval, respectively. Fig 5 shows the correlation coefficients of PM_2.5_ concentrations at each station at the current time and 1–24 hours, from which it can be seen that the autocorrelation coefficients of PM_2.5_ concentration values at all stations in 1–24 hours are 0.4 and above. Hence, it is feasible to predict the future PM_2.5_ concentration values to the results with the 24-hour values. However, as time in-creases, the value for the autocorrelation coefficient decreases, and when it exceeds 24 hours, the temporal correlation of the data is weaker.

**Fig 6:**
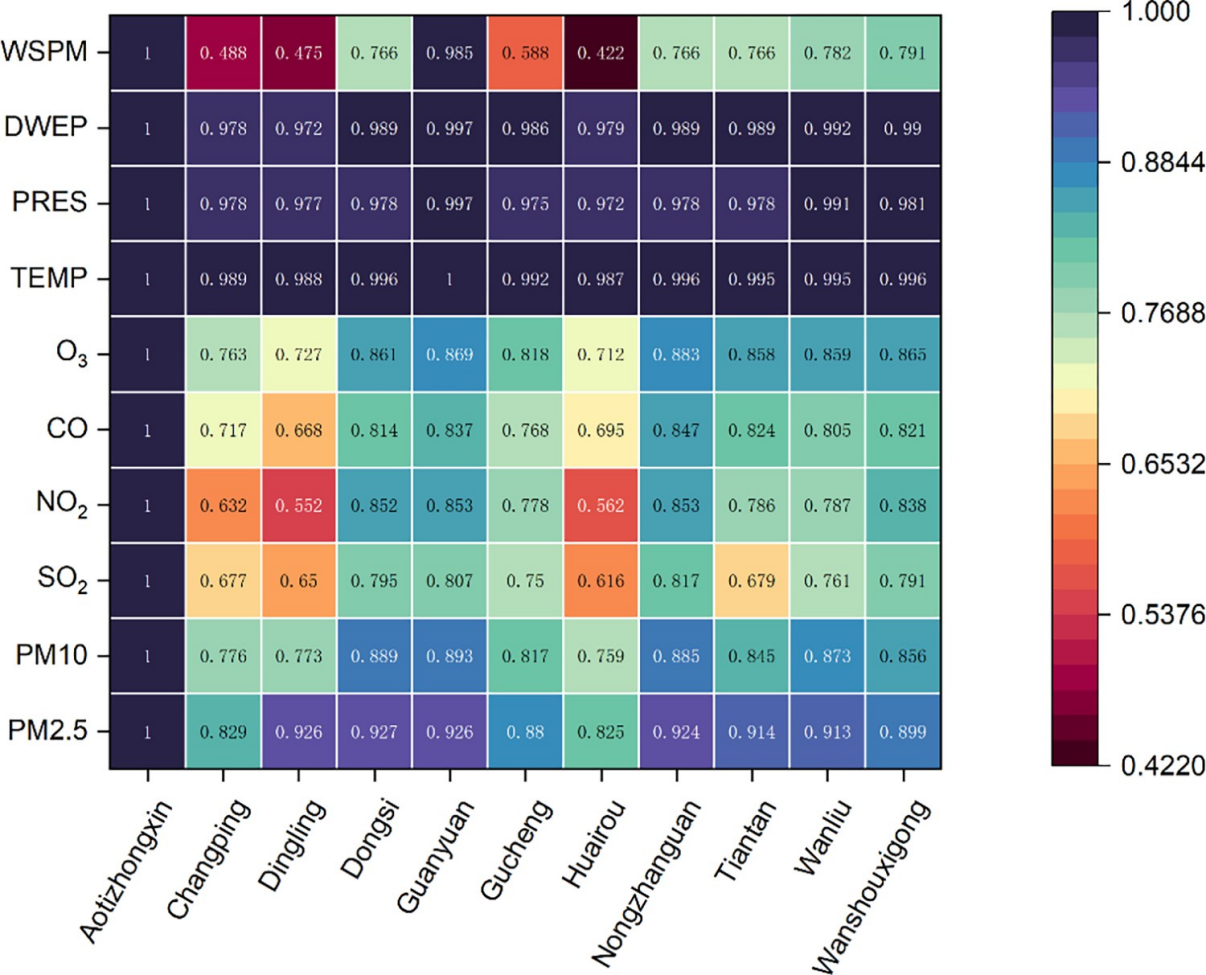
Spatial correlation analysis. Correlation coefficients of meteorological data and pollutant concentration data at each station.

There is a strong spatial correlation between air quality monitoring stations, and the Pearson correlation coefficient formula can also be used to calculate the correlation coefficients between the monitoring stations in the city. Fig 6 shows the heat map of the correlation coefficients between the central station of Aotizhongxin and the rest of the neighboring stations for each meteorological data and pollutant concentration data. From the heat map, it can be seen that the correlation coefficients between the neighboring stations and the central station are all greater than 0.42, the correlation between the central station and the neighboring stations is stronger, and the stations with larger correlation coefficients are used as neighboring stations to analyze the spatial correlation of the data.

### Spatiotemporal prediction model

#### Informer for forecasting

The Informer model, which effectively enhances the Long Sequence Time-Series Forecasting (LSTF) problem’s predictive power and confirms the potential utility of the Transformer-like model, is an improved version of the Transformer model. Three significant issues with Transformer are addressed by Informer: complexity in quadratic time and significant memory usage. The answer is suggested by the ProSparse Self-Attention Mechanism, self-attention distilling, and generative style encoder.

**Fig 7:**
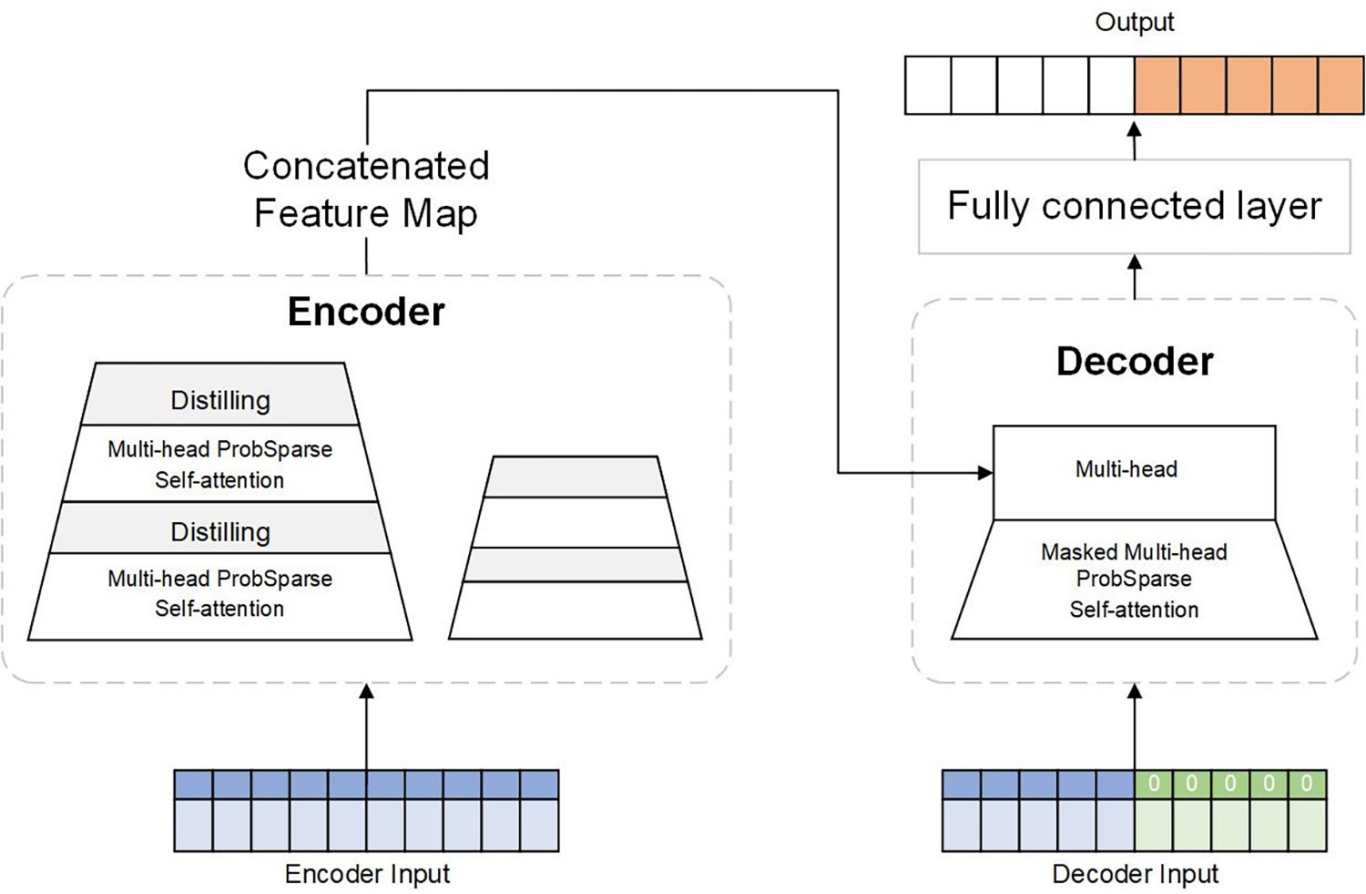
Model structure of Informer. Informer model maintains encoder-decoder structure, and it adds ProbSpare self-attention mechanism, which effectively reduces time complexity and memory usage.

Fig 7 depicts the Informer framework. A supervised learning model built on an attention process is known as an informer model. Encoder and decoder are the two components that make up the entire model. To save memory and expedite reasoning, the model enhances the Transformer-based self-attentive mechanism.

In the scrolling prediction setting with fixed window size, the input at time point t is

$$x^{t}=\{X_{1}^{t},\ldots ,X_{L_{X}}^{t}X_{d}^{t}\in R^{d_{x}}\}$$ (3)

The output predicts the corresponding sequence as

$$y^{t}=\{Y_{1}^{t},\ldots ,Y_{L_{y}}^{t}Y_{d}^{t}\in R^{d_{y}}\}$$ (4)

1. Encoder

The architecture of the Informer’s encoder for removing reliable remote dependencies from inputs for long-term series. The extra-long incoming data is given to the encoder component, as seen in Fig 7. The ProbSpare Self-Attention layer is used in lieu of the traditional Self-Attention layer in the encoder portion, and feature compression is then carried out by the Self-Attention distilling operation. The encoder module increases the robustness of the algorithm by stacking the two processes mentioned above.

The temporal complexity of self-attention dot product computation from optimization is resolved by ProbSpare Self-Attention. Instead of selecting the total dot product, the dot product computation is carried out by sampling each individual point. Allowing each person to concentrate solely on the main allows for ProbSparse self-attention. The equation reads as follows

$$A(Q,K,V)=softmax(\frac{\bar{Q}K^{T}}{\sqrt{d}})V$$ (5)

Where *Q*, *K*, and *V* are three matrices of the same size obtained by a linear transformation of the input feature variables, respectively $\left(Q\in R^{L_{Q}\times d},K\in R^{L_{K}\times d},V\in R^{L_{V}\times d}\right)$. *d* is a input dimension; $\bar{Q}$ is obtained from *Q* by probabilistic sparsification; Softmax is the activation function.

Self-Attention distilling is added as an improvement to the encoder for reducing the size of feature maps. the Self-Attention distilling operation is used for topic compression as a natural consequence of ProbSparse Self-attention, the encoder’s feature mapping brings about a V redundant combinations, using distilling to privilege dominant features with dominant features, and generating focus self-attention feature maps at the next layer.

2. Decoder

The decoder gets a long sequence of inputs and places a zero in the predicted target position. The final stage then involves passing through the Attention layer of the Mask to produce the anticipated output. The decoding process is described above, in which each decoder layer conducts feature extraction operations in the target’s direction based on the input.

A two-layer stack of Multi-head attention mechanism levels makes up the decoder. Because the future information is unknown at the time of generation, the first layer, Masked Multi-head ProbSparse self-attention, partly masks it out. The current output is then computed using only the prior data using the ProbSparse self-attention method. Multi-head ProbSparse self-attention is the second phase.

The Decoder gets a long sequence of inputs and places a zero in the projected target point. Then the predicted output is generated in the last step by passing through the Attention layer of the Mask. Each decoder layer performs feature extraction operations in the direction of the target based on the given input, and the above process is the decoding process. The decoder uses the encoder’s output data to determine what the current decoding should produce in the second layer, which is known as Multi-head ProbSparse self-attention.

#### ST-Informer

**Fig 8:**
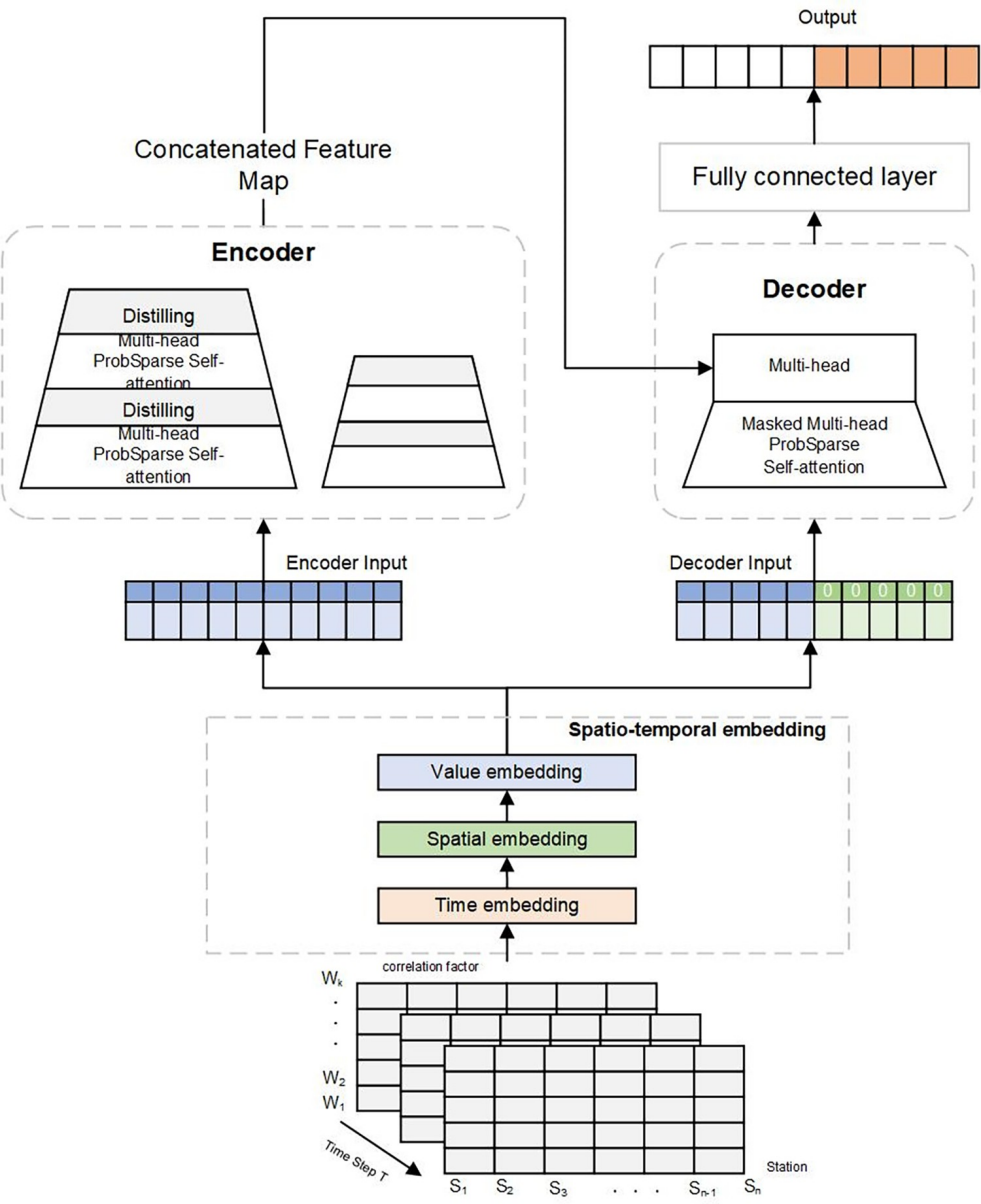
Structure diagram of ST-Informer model. A spatio-temporal embedding layer is added to the original Informer model to fully analyze the spatiotemporal characteristics of the input data.

In this paper, an improved model based on Informer is used to capture the spatiotemporal correlation of the data. As shown in Fig 8, the structure of the ST-Informer model is illustrated. In the constructed prediction model, the spatiotemporal data of a region over a while is collected as the input to the prediction task. The input data is a two-dimensional spatiotemporal matrix *X*(*X*∈*R*^*T*×*S*×*W*^), where *T* represents the time lag, and *S* means the air quality monitoring station information. *W* represents the air pollutants and meteorological characteristics. The input data is first input to the temporal embedding layer to extract the temporal correlation of the data fully. The temporal embedding layer uses the location embedding in the Transformer model to fully extract the temporal properties of this data. The spatial embedding layer directly captures the spatial characteristics of the input data using the multi-headed self-attentive mechanism. The addition of the spatial embedding layer captures not only the data’s static elements but also the data’s dynamic spatial dependencies. The value embedding layer separates different timestamps to compute correlations between sites and uses the correlations as weights to adjust the high-dimensional representation of all site input variables. Thus, the complex interdependencies between variables and their dynamic relationships are highlighted in the model. The spatial embedding layer, temporal embedding layer, and value embedding layer data are then linked to represent a comprehensive embedding feature. In the spatiotemporal embedding layer module, the main processes implemented in each layer are.

1. Temporal embedding layer

The model can represent temporal information thanks to the addition of a temporal embedding layer. Since the data’s reliance on time is complex, we must embed not only the sequence’s own temporal characteristics but also its position at the time of the input sequence. Positional coding must be used to embed the data’s temporal properties while taking into consideration the time series’ inherent periodicity.

$$\{\begin{array}{c} PE(pos,2i)=\sin(\frac{pos}{1000^{\frac{2i}{d_{model}}}})\\ PE(pos,2i+1)=\cos(\frac{pos}{1000^{\frac{2i}{d_{model}}}}) \end{array}$$ (6)

*d*_*model*_ refers to the dimensionality of the data after mapping through the input layer; *pos* refers to the position of a mapped feature in a row of data in the input data; 2*i* and 2*i*+1 denotes the number of rows of data; Eq (6) converts the data’s temporal order into a vector by mapping each series’ integer index to a high-dimensional representation. The input to the final time embedding layer is produced by extending each time features into a vectorized index for each new timestamp. The time embedding layer functions as a lookup table, storing embeddings in fixed hours, days, weeks, and months.

2. Spatial embedding layer

Using a multi-headed self-attentive mechanism, the spatial embedding layer immediately captures the spatial characteristics of the input data. Each air quality tracking station’s data has characteristics of spatial and temporal correlation, and by adding a spatial embedding layer, it is possible to record both the static and dynamic spatial dependence of the data.

The input values (*N*_*S*_×*N*_*V*_) at each moment are first projected into a high-dimensional subspace utilizing a feedforward neural network.


$$Z_{t}=w*X_{t}+b$$ (7)

*Z*_*t*_ is a high-dimensional representation of the input variables at moment *t*. Using the above equation, the site *i* and site *j* correlation strength *S*_*t*,*ij*_ can be calculated using the mutual covariance function as follows.


$$S_{t,ij}=xcorr(Z_{t,i},Z_{t,j})$$ (8)

*S*_*t*_ is obtained as a square matrix of *N*_*S*_×*N*_*V*_, representing the strength of the correlation between the two sites at moment *t*. The value of the spatial embedding layer output *E*_*S*_ at a given moment is calculated as

$$E_{S}=w*S_{t}*Z_{t}+b$$ (9)

3. Value embedding layer

The value embedding module uses a one-dimensional convolution of the input dimension *d*_*model*_ to represent the input sequence in high dimension as *E*_*V*_. The value embedding layer calculates the correlation between sites by separating the different timestamps and uses the calculation results as weights to adjust the high-dimensional representation of all site input variables. The value embedding does not separate timestamps. It makes use of the whole input time series and turns it into a single, highly dimensional representation matrix to illustrate the intricate relationships and dynamics between the model’s variables. A full embedding feature is then represented by connecting the spatial, temporal, and value embedding layer data

$$X_{ST}=E_{T}⊕E_{S}⊕E_{V}$$ (10)

The encoder-decoder of the Informer model is then fed data from the value encoding layer of the outputs. The Transformer model’s initial self-attention mechanism is replaced by a multi-head probabilistic sparse self-attention mechanism in the encoder, which significantly reduces the computational scale of the model and boosts robustness through layer-by-layer superposition. By padding the target prediction data to zero, measuring the feature map’s weighted attention composition, and then going through a hidden multi-head probability sparse self-attention mechanism layer, the decoder produces the prediction output.

## Results

### Performance of ST-Informer

The research model in this article was built using the meteorological data and pollutant concentration data introduced in Beijin, with 80% of the data serving as the training set, 10% as the test set, and 10% as the validation set. Following preprocessing, the tested outliers are marked as missing values in the collected dataset, and the KNN algorithm is then used to fill in the missing values.

The constructed prediction model of spatiotemporal data is used to predict the future PM_2.5_ concentration values at the central location using the collected 24 hours of spatiotemporal data of a particular area as the input sequence of the prediction task. By adjusting various batch sizes, learning rates, and iterations, the ST-Informer model’s training was used in this study to identify the ideal set of model parameters. The parameters chosen for this model in this exercise are shown in Table 3. The Mean Absolute Error (*MAE*), the Root Mean Square Error (*RMSE*) and the Coefficient of determination (*R*^2^)are then selected as the evaluation indicators of the model in this study. If the *MAE* and *RMSE* are smaller and the *R*^2^ is closer to 1, it means that the model has higher prediction accuracy.


$$MAE=\frac{1}{n}\sum_{i=1}^{n}\left|X_{act}(i\right)-X_{pred}(i)|$$ (11)

$$RMSE=\sqrt{\frac{\sum_{i=1}^{n}{\left[X_{act}(i)-X_{pred}(i)\right]}^{2}}{n}}$$ (12)

$$R^{2}=\frac{\sum_{i=1}^{n}{\left[X_{pred}(i)-\bar{X}\right]}^{2}}{\sum_{i=1}^{n}{\left[X_{act}(i)-\bar{X}\right]}^{2}}$$ (13)

in which *n* is the sample size of the test set; *X*_*act*_(*i*), *X*_*pred*_(*i*) are the true and predicted values of PM_2.5_ concentration at moment *i* (*i* = 1,2,…,*n*); $\bar{X}$ is the average of the true values of the predicted samples.

**Table 3:** Setting of ST-Informer model parameters.

attribute name	parameter setting
Encoder stack	3\2\1
Batch size	128
training cycle	20
Dropout	0.01
optimizer	Adam
learning rate	0.0001
activation function	Relu
loss function	MSE

### Model comparison

Three models were chosen by the experiment for comparison with the ST-Informer model used in this research in order to assess the models’ performance.

1. **LSTM:** LSTM is an RNN model with advantages over traditional neural network frameworks and is often used for time series prediction; and adds a memory cell to determine whether the information is valid, solving the gradient vanishing and gradient explosion problem during training of long sequences. This improvement allows it to perform better in longer sequences.
2. **Transformer:** The Transformer model is essentially a model built based on the Attention mechanism. Attention can solve the long-range dependency problem of RNN and its variants and supports parallelized computation.
3. **Informer:** Informer is an improved model based on Transformer that is more suitable for long sequence prediction. Informer aims to improve self-attentive mechanisms, reduce memory use, and speed up inference.

**Fig 9:**
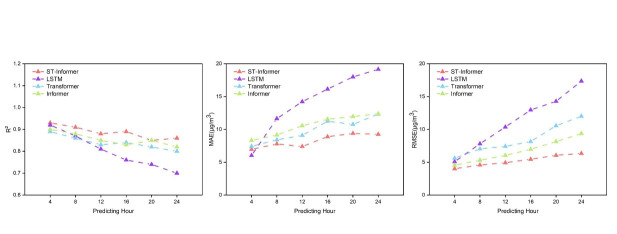
The value of the model evaluation index. The values of the evaluation indicators obtained by predicting PM_2.5_ in different models.

The above three models were selected in order to verify the advantages of this model in long-term prediction, we selected different times for PM_2.5_ concentration prediction. The above four models were used to predict PM_2.5_ concentrations for the next 4 hours, 8 hours, 12 hours, 16 hours, 20 hours, and 24 hours, respectively. The optimal set of hyperparameters was selected for each prediction model, and the results obtained were calculated for the evaluation indexes shown in Table 4. Fig 9 shows the specific values of each evaluation index for predicting different times in the future, and it can be seen in the figure that all four models’ show good results in predicting PM_2.5_ concentrations at 4 hours, with the value of *R*^2^ above 0.85. As the prediction length increases, the *MAE* and *RMSE* of all four models increase and the value of *R*^2^ decreases. This proves that the longer the prediction time is, the error of the model increases and the prediction becomes worse. the values of *MAE* and *RMSE* of the LSTM model increase significantly with the increase of the prediction time compared to the other models, and the difference between the predicted and the actual values is larger. The prediction accuracy of the Transformer and Informer models is lower than that of the ST-Informer model, and the ST-Informer model does not have a sudden decrease in prediction accuracy due to the long prediction sequences. The ST-Informer model has better prediction results than the other models mentioned above for longer sequences.

**Table 4:** Compare the performance of different models.

Model	MAE(μg/m^3^)	RMSE(μg/m^3^)	R^2^
ST-Informer	7.50 ± 3.16	4.31 ± 2.04	0.88 ± 0.05
LSTM	12.50 ± 7.41	10.34 ± 6.65	0.80 ± 0.12
Transformer	9.34 ± 5.66	7.89 ± 4.21	0.84 ±0.06
Informer	8.91 ± 3.57	6.52 ± 3.06	0.86 ± 0.04

**Fig 10:**
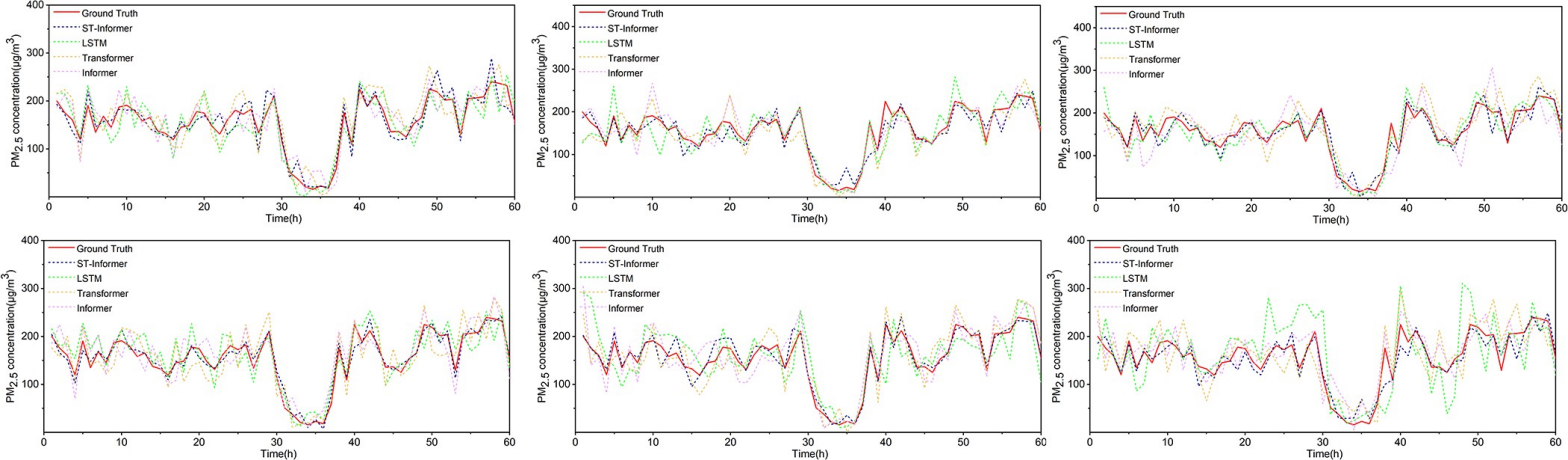
The results of four models to predict PM_2.5_ concentration in different time periods in the future. (a) The results of four models predicting PM_2.5_ concentrations over the next 4 hours; (b) The results of four models predicting PM_2.5_ concentrations over the next 8 hours; (c) The results of four models predicting PM_2.5_ concentrations over the next 12 hours; (d) The results of four models predicting PM_2.5_ concentrations over the next 16 hours; (e) The results of four models predicting PM_2.5_ concentrations over the next 20 hours; (f) The results of four models predicting PM_2.5_ concentrations over the next 24 hours.

To more visually show the advantages of the ST-Informer model in prediction, Fig 10 shows the prediction results of the four models for PM_2.5_ for the next 4 hours, 8 hours, 12 hours, 16 hours, 20 hours, and 24 hours, respectively. The PM_2.5_ concentrations were predicted from 0:00 a.m. on January 1 to 12:00 a.m. on January 3, 2017 (a total of 60 hours), and it can be seen from the figure that the predicted PM_2.5_ concentrations using the ST-Informer model are closer to the true values, and the model can more fully extract the spatial and temporal characteristics of the data. As the predicted future PM_2.5_ concentrations increase in time, the ST-Informer model shows better performance, and the model can capture the sudden changes in the predicted values more accurately and has better prediction results.

### Application of pollutant concentration prediction

**Fig 11:**
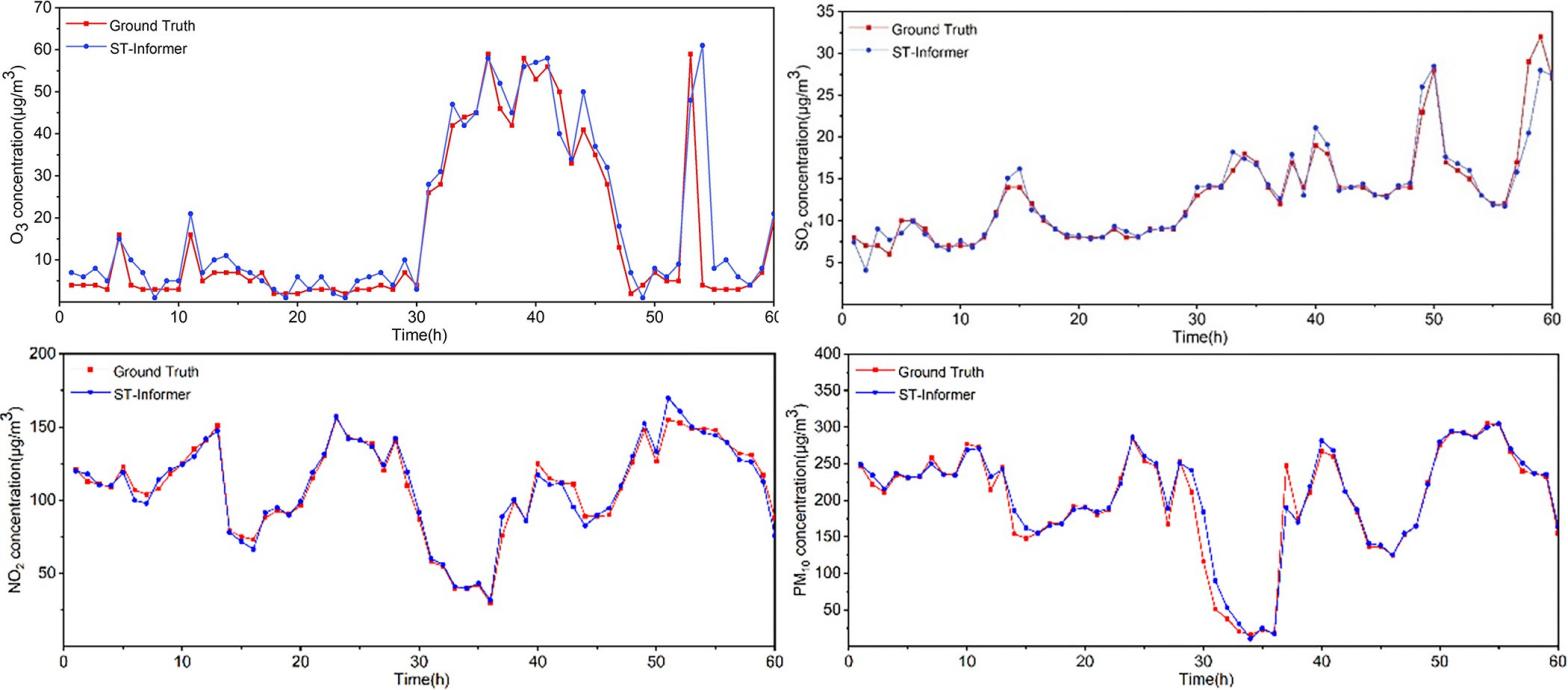
Predicted results of other air pollutant concentrations. The ST-Informer model was used to predict 60-hour other air pollutant concentrations. (a) The results of the O_3_ concentration prediction. (b) The results of the SO_2_ concentration prediction. (c) The results of the NO_2_ concentration prediction. (d) The results of the PM_10_ concentration prediction.

To further analyze the generalizability of the ST-Informer model, the model was used to predict the concentration values of other pollutants. This paper selected four atmospheric pollutants, O_3_, SO_2_, NO_2_, and PM_10_, as targets for prediction. O_3_, SO_2_, NO_2_, and PM_10_ are also significant components of atmospheric pollutants, and these pollutants are also extremely harmful to the environment [34–36], so predicting the concentrations of these pollutants also has important practical significance. In past studies, many scholars contended that the prediction of these four pollutants had been studied separately, and all of them have achieved good prediction results. In this paper, in investigating the generalizability of the ST-Informer model, the model was trained with the four pollutant concentrations as the output of the model, and the optimal parameter set of the model was selected. The final prediction results of the model are shown in Fig 11. It can be seen that the ST-Informer model also achieves good results in the prediction experiments of other pollutant concentrations. It is proved that the ST-Informer model can be used to solve various complex spatiotemporal prediction problems.

## Discussion

This study aims to combine deep learning models to develop a prediction model for spatial and temporal data, through which PM_2.5_ concentrations can be accurately predicted to provide data support for relevant departments for PM_2.5_ management. Specifically, this paper does the following work: (1) Taking Beijing as an example, meteorological data and pollutant concentration data from several stations in the city are integrated. And the data set is finely pre-processed to make sufficient preparation for the input of the subsequent prediction model and effectively improve the prediction ability of the model. (2) The multidimensional historical meteorological data and pollutant concentration data were analyzed for spatial and temporal correlation, and the dynamic multivariate dependence of the data in space and time was analyzed. (3) Capturing the highly dynamic spatiotemporal dependence in the data. A SpatioTemporal-Informer (ST-Informer) model is proposed, which utilizes independent temporal and spatial embedding layers to process the input data and capture the complex spatiotemporal properties of the data, and the model’s unique ProbSpare Self-Attention mechanism is used to focus on extracting data across time, spatial and multidimensional information of the data. In this study, the research results in the attention mechanism are applied to the spatiotemporal data prediction task, and a novel PM_2.5_ concentration prediction model combining spatial location, temporal variation and contextual information is proposed, which provides a new idea for PM_2.5_ concentration prediction.

In the process of verifying the performance of the ST-Informer model, three more typical deep learning models, LSTM, Transformer and Informer, were selected for comparative analysis. The mean absolute error MAE, root mean square error RMSE and coefficient of determination R^2^ were selected as the evaluation indexes of the models in this study. According to the experimental results, the ST-Informer model has higher prediction accuracy compared with the other three comparison models when predicting PM_2.5_ concentrations of different time durations. In the face of sudden and abrupt changes in PM_2.5_ concentration values, the other comparison models tend to produce erroneous analyses and the prediction results have large errors with the true values, while the ST-Informer model can more sensitively sense large changes in the predicted data. Secondly, to study the generalizability of the ST-Informer model, this paper also applies the model to the prediction of other pollutant concentrations, such as O_3_, SO_2_, NO_2_, and PM_10_, and compared with the prediction of other pollutant concentrations, the real value of SO_2_ is smoother and the model effect is significant, while for the prediction of other pollutant concentrations, the prediction results of ST-Informer model are closer to the prediction results of ST-Informer model are also close to the real results.

The model still needs further improvement in the future: (1) In predicting PM_2.5_ concentrations, the influencing factors are not sufficiently considered, and the influence of factors such as geographical environment is not taken into account. (2) This study only predicted the PM_2.5_ concentration data in a few cities, and the migration analysis of the model is not comprehensive enough. In future work, the ST-Informer model can be applied to predict pollutant concentrations in multiple regions. In addition, the model can also be applied to other studies of spatiotemporal data prediction.

## Conclusion

This study proposes a spatiotemporal prediction model: SpatioTemporal-Informer (ST-Informer), which is used to cope with the accurate prediction of fine particulate matter PM_2.5_ in the air. The model introduces a spatiotemporal embedding layer, which calculates the temporal, spatial, and value embedding layers separately and analyzes the data temporal and spatial correlations independently. In addition, the model applies the ProbSparse Self-Attention mechanism, which may contribute to a more focused analysis of the complex dynamic spatial and temporal variation characteristics of PM_2.5_ concentrations. In this paper, the ST-Informer model is applied to predict PM_2.5_ concentration at a site in Beijing. The selected influencing factors include meteorological factors and air pollutant concentration factors. The historical data of adjacent areas are used to predict the future PM_2.5_ concentration values, and the ideal prediction results are achieved. In the experiments of predicting PM_2.5_ concentration, the prediction results of the ST-Informer model proposed in this paper are significantly better than those of the LSTM, Transformer, and Informer models $\left(MAE\approx 7.50\mu g/m^{3},RMSE\approx 4.31\mu g/m^{3},R^{2}\approx 0.88\right)$. In addition, the ST-Informer model can more thoroughly analyze the nonlinear relationship between the data and more sharply detect sudden changes in PM_2.5_ concentration values and identify the peaks of the data. In addition, the model is more generalizable and has been applied to predicting other pollutant concentrations with good results. The ST-Informer model can be applied to prediction problems with complex spatial and temporal characteristics and can also effectively identify peak spikes or pits due to various factors. The results of this study provide data support for the national control of air pollution. Relevant departments can make corresponding air pollution control measures based on the results of this study according to local conditions.
